# Colorectal cancer vaccines: The current scenario and future prospects

**DOI:** 10.3389/fimmu.2022.942235

**Published:** 2022-08-03

**Authors:** Wenqing Jia, Tao Zhang, Haiyan Huang, Haoran Feng, Shaodong Wang, Zichao Guo, Zhiping Luo, Xiaopin Ji, Xi Cheng, Ren Zhao

**Affiliations:** ^1^ Department of General Surgery, Ruijin Hospital, Shanghai Jiao Tong University School of Medicine, Shanghai, China; ^2^ Shanghai Institute of Digestive surgery, Ruijin Hospital, Shanghai Jiao Tong University School of Medicine, Shanghai, China

**Keywords:** colorectal cancer, vaccine, neoantigen, nanovaccines, immunotherapy

## Abstract

Colorectal cancer (CRC) is one of the most common cancers worldwide. Current therapies such as surgery, chemotherapy, and radiotherapy encounter obstacles in preventing metastasis of CRC even when applied in combination. Immune checkpoint inhibitors depict limited effects due to the limited cases of CRC patients with high microsatellite instability (MSI-H). Cancer vaccines are designed to trigger the elevation of tumor-infiltrated lymphocytes, resulting in the intense response of the immune system to tumor antigens. This review briefly summarizes different categories of CRC vaccines, demonstrates the current outcomes of relevant clinical trials, and provides particular focus on recent advances on nanovaccines and neoantigen vaccines, representing the trend and emphasis of CRC vaccine development.

## 1 Introduction

Colorectal cancer (CRC) is one of the most common cancers worldwide and accounts for nearly 8.5% of total cancer mortality (1). Radical surgical resection, chemotherapy, radiotherapy, and targeted therapy are the main treatment approaches for CRC (2). Immune checkpoint blockade (ICB) therapy, such as pembrolizumab and nivolumab, targeting programmed death 1 (PD-1), has been approved for the treatment of CRC that is mismatch repair deficient (dMMR) or has high microsatellite instability (MSI-H) (3, 4). However, MSI-H CRC patients account for only about 15% of the total, and the other 85% are mismatch repair proficient (pMMR), that is, have tumor microsatellite stability (MSS), which are not sensitive enough to existing treatment (5).

This phenomenon may be related to the preexistence of tumor-infiltrating lymphocytes (TILs) in MSI-H patients due to higher immunogenicity caused by the tumor mutational burden (TMB). In contrast, pMMR/MSS status with lower TMB triggers a slight immune response (6). Cancer vaccines could trigger an intense immune response to one or more specific antigens, enhancing local TIL infiltration, leading to cytotoxic effects to cancer cells expressing those antigens. Such a tumor-immune cycle starts with the administration of tumor vaccines containing specific tumor antigens, followed by activation of antigen-presenting cells (APCs), especially dendritic cells (DCs). Immature DCs demonstrate a strong capability of recognizing and capturing antigens through phagocytosis and micropinocytosis (7). After antigen uptake, major histocompatibility complex (MHC) I/II and costimulatory molecules on the surface of DCs will be upregulated due to the production of interleukin (IL)-12 and chemokines. The antigen-loaded DCs then migrate to draining lymph nodes, which are the primary site of T-cell activation. Mature DCs present the processed antigen epitopes on MHC I or MHC II to naive T cells, priming tumor-specific T cells through a two-signal process (8). Activated T cells yield both effector T cells and long-lived memory T cells (9). Effector tumor-specific T cells amplify and move through blood flow into the tumor microenvironment (TME) to induce tumor destruction through cytotoxicity and the production of certain cytokines [e.g., interferon (IFN)-γ and tumor necrosis factor (TNF)-α]. Also, CD4+ T-helper cells (Th1) in different compartments activate DCs through CD40/CD40L interaction and equip tumor cells with more MHC I on the surface by releasing IFN-γ, orchestrating various cell types and contributing to an inflammatory environment (10, 11). Additionally, activated B cells promote tumor killing effects through antibody-dependent cellular cytotoxicity (ADCC) (12). In turn, stressed tumor cells release vast numbers of antigens and damage-associated molecular patterns (DAMPs) that are captured, processed, and presented by APCs to induce polyclonal T-cell responses, thereby multiplying antitumor immune responses (13). Overall, cancer vaccines contribute to tipping the balance from tolerance toward active immunity against tumor cells, rendering the cancer immunity cycle functional.

Compared with traditional therapies, vaccines are generally well tolerated and almost with no dose-related toxicity (14). A great breakthrough in the development of cancer vaccines has been made in the last decade. With the development of sipuleucel-T, approved by Food and Drug Administration (FDA) in 2010, the cancer vaccine field has received massive attention and exploration (15). In the case of CRC, there has been an introduction of new cancer vaccines.

In this review, we summarize the development of appropriate antigens and different vaccine types and adjuvant delivery systems of CRC vaccine. Recent progress in the field in the past 3 years and the prospect of future development were also listed and discussed.

## 2 Tumor antigens in colorectal cancer vaccine development

**Figure 1:**
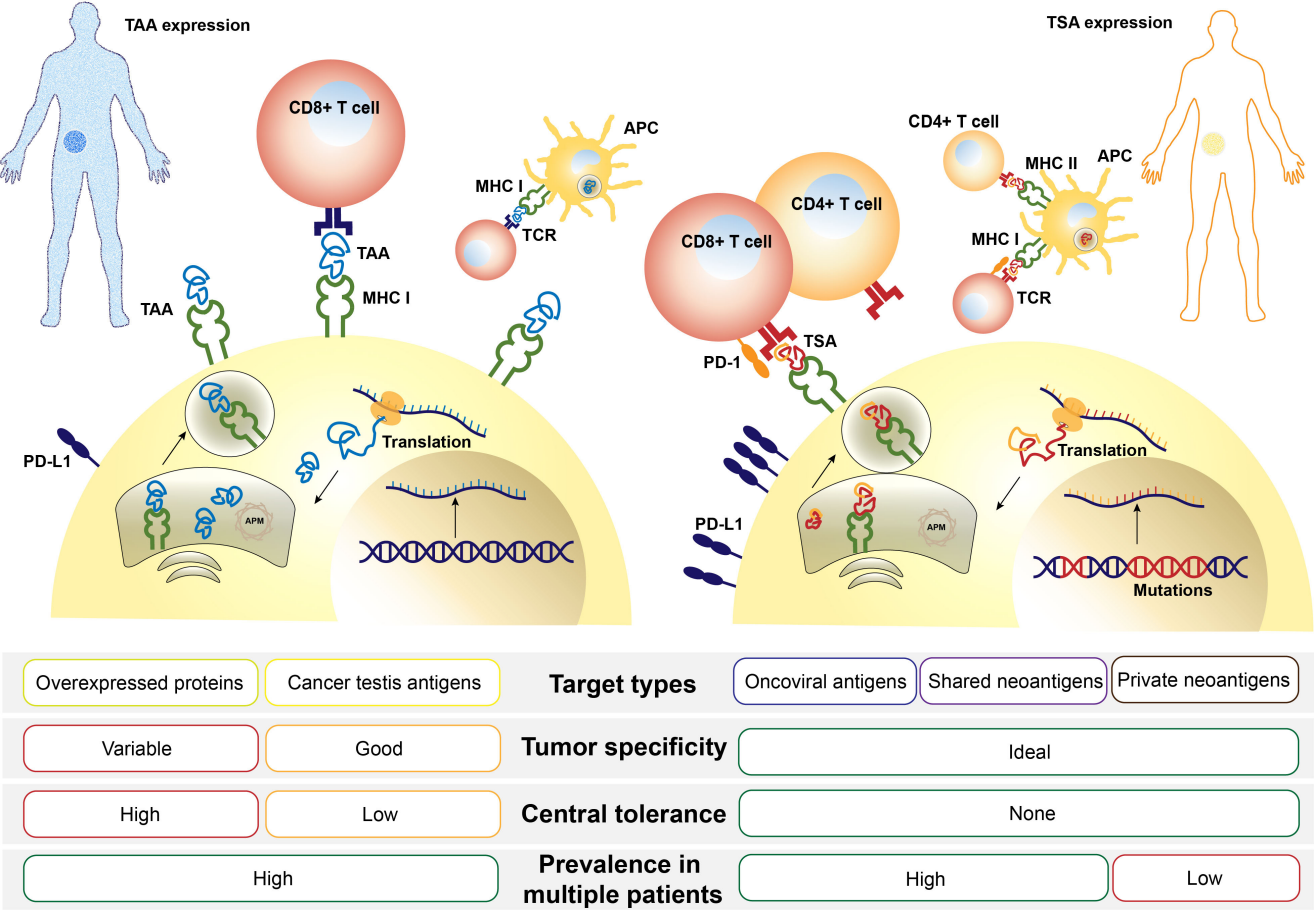
Comparison of tumor-associated antigens (TAAs) and tumor-specific antigens (TSAs). TAAs or TSAs are processed in an order depicted above, including transcription of a genomic locus (TAA) or mutation-containing locus (TSA), translation and posttranslation modification, protein degradation, and MHC molecule loading. After finally being presented on the cell surface, antigens are recognized by T cells *via* T-cell receptor (TCR) and a sequence of costimulation. APM, antigen-presenting machinery; MHC, major histocompatibility complex.

Determining the appropriate tumor antigen is an initial stage in the formulation of the CRC vaccine. Tumor antigens can be divided into two types: tumor-associated antigens (TAAs) and tumor-specific antigens (TSAs), also called neoantigens. The former are proteins overexpressed by tumor cells compared with normal cells, while the latter are expressed only by cancer cells but not by normal cells (16). They both can be presented by human leukocyte antigen (HLA) to T cells, initiating an immune response (**Figure 1**).

### 2.1 Tumor-associated antigen

Carcinoembryonic antigen (CEA) and melanoma-associated antigen (MAGE) are the first TAAs ever identified and widely explored in the clinical trials of CRC vaccine (16). Other TAAs targeted for CRC treatment include mucin 1 (MUC-1), epidermal growth factor receptor (EGFR), vascular endothelial growth factor receptor 1 and 2 (VEGFR1, VEGFR2), transmembrane 4 superfamily member 5 protein (TM4SF5), survivin, mitotic centromere-associated kinesin (MCAK), guanylyl cyclase C (GUCY2C), and 5T4 (17–20).

### 2.2 Tumor-specific antigen

TSAs, produced by cancer cells carrying mutations affecting protein sequences, include non-synonymous point mutations, indel mutations, frameshift mutations, splicing mutations, and gene fusion (2, 21). Several frequently presenting frameshift mutations include TGFβR II, HT001, TP53, AIM2, and mutant KRAS (22, 23).

Previous studies (24–28) identified novel TSAs in three steps: 1) identifying somatic mutations or productions in DNA or messenger RNA (mRNA) sequences; 2) evaluating the affinity and presentation of MHC I/II molecules with new peptides (29); 3) determining whether new epitopes could stimulate T-cell proliferation and related immune responses (2). By improving the algorithm (30) and exploring subtype-specific antigens (24, 27, 31), potential antigen targets of CRC vaccine are gradually found, which lay a foundation for subsequent vaccine preparation.

## 3 Different types of colorectal cancer vaccines

### 3.1 Molecular-based vaccine

**Figure 2:**
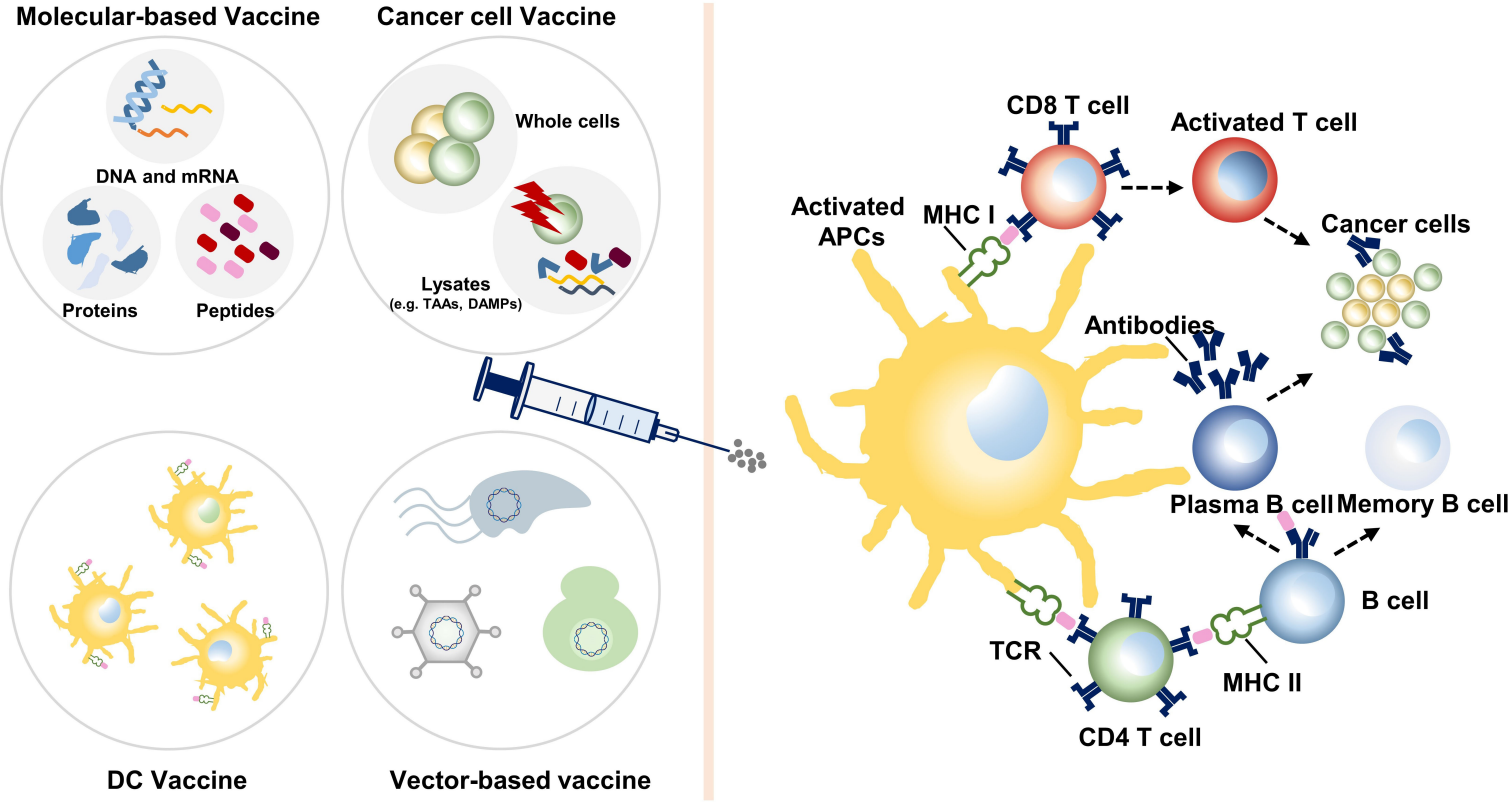
Various categories of colon cancer vaccines and their mechanisms. Dendritic cell (DC) vaccines utilize DCs loaded with tumor antigens *ex vivo* or transfected to express tumor antigens. Molecular-based vaccines and cancer cell vaccines stimulate the autologous antigen-presenting cells (APCs), most are DCs. Then, effector immune cells are activated, boosting an instant and long-term antitumor reaction. TAAs, tumor-associated antigens; DAMPs, damage-associated molecular patterns; MHC, major histocompatibility complex; TCR, T-cell receptor.

Molecular-based vaccines include peptide/full-length protein vaccine and DNA and mRNA vaccines (**Figure 2**).

The protein-based vaccine contains abundant immunogenic sites (including TAA or TSA), which can be processed and presented by MHC I/II epitopes. In a phase II trial involving 96 patients with advanced CRC, a vaccination mixture of five HLA-A*2404-restricted peptides (RNF43, KOC1, TOMM34, VEGFR1, VEGFR2) was proven safe while simultaneously applied with oxaliplatin-based chemotherapy (32). A combination of KOC1, TTK, URLC10, DEPDC1, and MPHOSPH1 as an HLA-A*2404-restricted vaccine also proved safe and presented with 9.4 months’ overall survival (OS) in a phase I trial (33).

DNA vaccines introduce gene sequences encoding tumor antigens into the body through plasmids. The products of transcription and translation are then presented by MHC I or II molecules. Additionally, the DNA structure can also activate innate immunity through cytoplasmic sensors (17). But more advanced manufacturing technology is urgently needed to produce vaccines that can be transported into the nuclear membrane. Meanwhile, the plasmid could be integrated into the host genome, which increases the uncertainty of products and efficiency.

The mRNA vaccine is the synthesis of RNA-encoding tumor antigens *in vitro*. After being internalized by the target cells, it completes the translation in the cytoplasm without entering the nuclear membrane. Compared with the DNA vaccine, mRNAs are more effective, easier to modify for different purposes, and the production of which is less time-consuming. mRNA-4157 is a Moderna mRNA-based cancer vaccine depicting safety and clinical efficiency when dosing combined with pembrolizumab in a phase I trial (NCT03313778) (34). A phase I trial of mRNA 5671, a vaccine against KRAS-positive cancers, combined with pembrolizumab in non-MSI-H patients is underway (NCT03948763) (**Table 1**).

**Table 1:** Major clinical trials of colorectal cancer vaccines.

	Interventions	Adjuvant/ combined therapy	Stage	Design and arms	Primary objective	Results	Phases	NCT Number/ Reference
**Moleculer-based vaccine**	HLA-A*2402-restricted peptides	mFOLFOX6 or XELOX	advanced CRC	Single arm: HLA-A*2402 restricted peptides + chemotherapy (N=96)	RR, PFS, OS	No significance was observed for planned statistical efficacy endpoints.	II	(32)
	Five HLA-A*2402-restricted peptides	Cyclophosphamide	advanced CRC	Single arm: Five HLA-A*2402-restricted peptides + chemotherapy (N=9)	Safety, immune response	The vaccine was safe. Induced T-cell responses were observed.	I	(33)
	mRNA 4157	Pembrolizumab	mCRC	Arm 1: Vaccine at different doses Arm 2: Vaccine + chemotherapy	Safety, immune response	A portion of results showed that this vaccine was safe, well-tolerated and could induce strong neoantigen-specific T cell responses.	I	NCT03313778 (34)
	V 941 (mRNA 5671)	Pembrolizumab	KRAS positive cancers	Arm 1: Vaccine alone Arm 2: Vaccine + chemotherapy	Safety, immune response, ORR	The clinical trial is underway and the results are eagerly awaited.	I	NCT03948763
**Cancer cell vaccines**	OncoVax	Bacillus Calmette-Guerin (BCG)	Stage II (N=297) , Stage III (N=115)	Treatment arm: Surgery + vaccination Observation arm: Surgery alone	OS, DFS	Trend toward better DFS (p = 0.078) and OS (p = 0.12). DFS (p = 0.006) and OS (p = 0.017) improved in stage II patients.	III	(35)
	GVAX	GM-CFS, cyclophosphamide/ Pembrolizumab	advanced pMMR CRC	Single arm: GVAX/Cy + pembrolizumab (N=17)	ORR, safety, PFS, OS, DOR	The median PFS was 82 days (95% CI 48-97days) and the median OS was 213 days (95% CI 179-441 days). Toxicities were acceptable.	II	NCT02981524 (36)
		GM-CSF, cyclophosphamide/ guadecitabine	advanced CRC	Single arm: GVAX/Cy (N=18)	Immune response, safety	No significant increase in CD45RO+cells was noted. Grade 3–4 toxicities were expected and manageable.	I	NCT01966289
**Dendritic cells vaccine**	Dendritic cells vaccine	/	mCRC	Vaccine arm: DC vaccine (N=8), Control arm (N=7)	DFS	DFS of the vaccine arm was 25.26 months (95% CI 8.73-n.r) versus 9.53 months (95% CI 5.32-18.88) in control arm.	II	NCT01348256 (37)
		*Ex vivo* TNF-α, IL1, IL6, and PGE2	advanced CRC	Single arm: DC vaccine (N=20)	Safety, OS, RFS	Median OS 7.4 m + G3 (G3 CI, 4.5–17.5 m); median time of tumor progression, 2.4 months (95% CI 1.9–4.1 months).	I/II	(38)

mCRC, metastatic colorectal cancer; MSS, microsatellite stable; pMMR, proficient mismatch repair; RR, response rate; PFS, progression-free survival; OS, overall survival; ORR, objective response rate; DFS, disease-free survival; DOR, duration of response; PFS, progression free survival.

### 3.2 Cancer cell vaccine

The cancer cell vaccine is an approach to use whole cancer cells or the lysates to prime the immune system (**Figure 2**). Based on the origin of cancer cells, cancer cell vaccines can be divided into autologous or allogeneic ones. Autologous vaccines are more specific to individuals, while allogeneic ones are more time-saving to produce, so as to benefit large-scale groups (39). The possibility of immune ignorance is reduced due to the large pool of unknown antigens. However, since it also contains many antigens widely expressed in normal tissues, cancer cell vaccine may induce certain autoimmune reactions.

OncoVax is one of the most widely studied CRC cancer vaccines with early phase clinical trials in 1980s (6). It is a combination of autologous cancer cells with bacille Calmette–Guérin (BCG) vaccine. Hoover et al. (40), Harris et al. (35), and Vermorken et al. (41) conducted several studies depicting significant effects of either three- or four-vaccination strategy of this vaccine as an adjuvant to surgery. The Eastern cooperative oncology group (ECOG) study 5383, a phase III trial, randomized 412 patients with CRC to be treated with surgery alone or surgery plus vaccination. After 7.6 years of follow-up, there was no significant difference in disease-free survival (DFS) or OS. Excitingly, subgroup analysis pronounced that stage II patients did have improved OS and DFS (OS p = 0.017; DFS p = 0.002) (35). To improve the clinical effects of OncoVax on stage III patients, its combination with 5-fluorouracil (5FU) and leucovorin proved to be a safe approach; furthermore, a randomized international phase III study is on the way (42).

GVAX is an allogeneic whole-cell vaccine modified to secrete granulocyte-macrophage colony-stimulating factor (GM-CSF). GVAX demonstrated a modulatory effect on the antitumor response in a phase II trial targeting pMMR advanced CRC patients (36). In order to further improve GVAX, epigenetic therapy has been tried to enhance immunologic activity in both preclinical and clinical trials (NCT01966289) (43, 44).

Numerous other types of cancer vaccines need further investigation in clinical trials, such as oncolytic virus (45–47) or immune cell death (ICD) (48), leading to *in situ* vaccine and colorectal cancer stem cell (CCSC) (49–51) as source of massive antigens.

### 3.3 Dendritic cell vaccine

DCs, isolated by leukapheresis, are one of the most effective APCs in the human body (**Figure 2**). DCs are matured in culture with cytokines and pulsed with exogenous peptide or tumor lysate to be prepared for infusion into patients. DC vaccines, indicating ideal effects in clinical trials of melanoma and prostate cancer (52), are commonly studied in CRC as well.

Rodriguez et al. (37) conducted an Randomized Control Trial (RCT) involving patients with surgically amenable liver metastasis of CRC (n = 19). Fifteen patients were randomly divided into two groups, receiving DC vaccinations or observation after surgery and chemotherapy. Median DFS of the vaccine arm and observation arm was 25.26 months (95% CI 8.74–not reached (n.r.)) and 9.53 months (95% CI 5.32–18.88), respectively (37).

MelCancerVac, a vaccine consisting of DCs, is generated by pulse of an allogeneic melanoma cell lysate from DDM-1.13 for its high expression of MAGE-A3, which is also a TAA overexpressed in CRC (6). Twenty patients with stage IV CRC were involved in a phase II trial, receiving up to 10 intradermal vaccinations biweekly. Although the overall results of the trial did not show a large improvement in OS (median OS 7.4 months), five patients experienced prolonged progression-free survival (PFS) (>6 months), two of which remained progression-free for >27 and >37 months (38). A further phase III trial of MelCancerVac for CRC patients is planned, and the results are expected in the future.

To improve the DC vaccine, more research is needed to compare different subsets such as monocyte-derived DCs (moDCs), conventional DCs (cDCs), plasmacytoid DCs (pDCs) (7). Induced pluripotent stem cells (iPSCs) may also be a choice (53).

### 3.4 Vector-based vaccine

Biological vectors include viral vectors, live-attenuated bacteria, and yeasts. They can be modified to express specific cancer antigen transgenes and initiate an immune response through pathogen-associated molecular patterns (PAMPs)–pattern recognition receptors (PRRs) interaction (54). The comparative safety, immunogenicity, and efficacy profile of vector-based vaccines are provided based on the evidence from CRC clinical trials (**Table 2**).

**Table 2:** Major clinical trials of biological vector-based cancer vaccines.

	Interventions	Carrier/source	Stage	Design and arms	Primary objective	Results	Phases	NCT Number
**Viral vector-based vaccine**	Ad5 [E1-, E2b-]- CEA(6D)	ADV	advanced CRC	Single arm: Ad5 [E1-, E2b-]-CEA(6D) (N=32)	Safety, immune response	There was minimal toxicity, OS at 12 months is 48%.	I/II	NCT01147965 (55)
	Ad5-hGCC-PADRE vaccine	ADV	stage I/II	Single arm: Ad5-hGCC-PADRE vaccine (N=10)	Safety, immune response	GUCY2C-specific T-cell responses were detected in 40% of patients. Adverse events were minimal.	I	NCT01972737 (56)
	PANVAC	poxvirus	mCRC	Arm 1: PANVAC + GM-CSF Arm 2: PANVAC + DCs	OS, RFS	2-year RFS (55% vs. 47%, p=0.22)	II	(57)
	Therapeutic autologous dendritic cells	PANVAX (viral vector)	mCRC	Vaccine arm: DC+PANVAC (N=39) Control arm: DC+GM-CSF(N=37)	RFS, immune response	RFS at 2 years was similar (47% and 55% for DC/PANVAX and PANVAX/GM-CSF, respectively). Specific T-cell responses against CEA was statistically similarly.	II	NCT00103142
	TroVax	MVA	mCRC	Single arm:TroVax (N=22)	Safety, immune response	Toxicity was minimal. Antigen-specific responses were observed.	I/II	(58)
	AVX701 (VRP-CEA(6D))	alphavirus	Stage III-IV	Arm 1: AVX701 (Stage IV, N=28) Arm 2: AVX701 (Stage III, N=12)	OS, RFS, immune response	Stage IV group: 5-year OS 17% (95% CI 6% to 33%) Stage III group: 5-year RFS 75% (95% CI 40% to 91%). An increase in CD8+TEM and a decrease in FOXP3+Trges were observed in 10/12.	I	(59)
**Live-attenuated bacteria and yeast vaccines**	GI-6207 (Yeast-CEA)	heat-killed yeast (*Saccharomyces cerevisiae*)	mCRC	Single arm: GI-6207 (N=22)	Safety, immune response	GI-6207 vaccination had minimal toxicity and induced certain antigen-specific T cell responses and CEA stabilization in patient population.	I	NCT00924092 (60)
	GI-6301 (Yeast Brachyury Vaccine)	yeast	advanced CRC	Single arm: GI-6301 at different dose levels (N=11)	Immune response, safety	Brachyury-specific T-cell responses was seen in the majority of patients. No evidence of autoimmunity or serious adverse events was observed.	I	NCT01519817 (61)
	GI-4000 ( whole, heat-killed, recombinant Saccharomyces cerevisiae yeast, engineered to encode *ras* oncogene)	yeast	advanced CRC (with specific *ras* mutation)	Single arm: GI-4000 (N=19)	Safety, immune response	GI-4000 demonstrated a favorable safety profile and immunogenicity in the majority of subjects.	I	(62)

ADV, adenovirus; MVA, modified vaccinia Ankara; OS, overall survival; RFS, recurrence-free survival; mCRC, metastatic colorectal cancer.

#### 3.4.1 Viral vector-based vaccines

Highly transfected viruses are mainly composed of adenoviruses, poxviruses, and lentiviruses. Adenovirus subtype 5 (Ad5)-based vectors with deletions of the E1 and E2b regions are designed to overcome host immunity after repeated exposure to Ad5 (63). Morse et al. (55) conducted Ad5 [E1-,E2b-]-CEA (6D) to enhance CEA-specific T cell-mediated immune response and proved its safety and efficacy in 32 metastatic colorectal cancer (mCRC) patients recruited to a phase I/II trial with OS of 48% at 12 months. E1/E3-deleted Ad5 inserted with GUCY2C and PADRE sequences proved safe in a phase I trial (56), and a phase IIa trial is still under exploration (NCT04111172).

PANVAC is a combination of poxvirus platform inserted with genes of CEA and MUC-1, along with TRICOM. A prime-boost strategy, PANVAC-V/F, is most often used to decrease neutralized antibodies (64). Gulley et al. (65) proved its tolerance in a phase I study, while Morse et al. (57) conducted a multicenter trial comparing effects of PANVAC plus GM-CSF or PANVAC-modified DCs in 74 postoperative mCRC patients who underwent adjuvant chemotherapy (90% OS at 40 months), showing no significant in 2-year Recurrence-free survival (RFS) (55% vs. 47%, p = 0.22).

Other vaccines with intrinsic outcomes in phase I/II clinical trials include TroVax (modified vaccinia Ankara encoding 5T4 antigen) (58, 66), ALVAC-CEA-B7 (avipox expressing CEA and B7.1) (67, 68), and AVX701 (a virus-like replicator particle containing CEA) (59, 69).

#### 3.4.2 Live-attenuated bacteria and yeast vaccines

As recombinant vaccine vectors, it is essential to segregate bacteria’s immunogenicity from their toxicity before manufacturing. Strategies for attenuating bacterial virulence include diminishing the replication capacities, suppressing virulence factor expression, and providing killed but metabolically active bacteria (70). Various live-attenuated bacterial platforms have been developed in preclinical studies for the treatment of CRC (17).

Attenuated strains of *Listeria monocytogenes* have been utilized as vaccine vectors targeting different tumors, especially human papillomavirus (HPV)-associated cancers, pancreatic cancer, and malignant pleural mesothelioma, improving the survival of patients (71). As for CRC, a personalized live-attenuated, double-deleted (pLADD) *L. monocytogenes*-based immunotherapy was designed for a phase 1 trial to analyze its safety (NCT03189030). Furthermore, ADX-NEO, a combination of *L. monocytogenes* platform and neoantigens, is undergoing a phase I trial in patients with metastatic solid tumor (NCT03265080). Although *L. monocytogenes* vaccines have demonstrated poor CD8+ T-cell priming for GUCY2C (72), their combination with Ad5.F35 vaccine against GUCY2C demonstrated robust expansion of specific T cells (73). The PeptiBAC tumor vaccine platform composed of BCG is easy to customize into a personalized cancer vaccine and deserves further investigation (74).

Heat-killed *Saccharomyces cerevisiae* as a vector, encoded with CEA or TSA to form GI-6207 (60), GI-4000 (62), and GI-3601 (61), proved safe in several phase I trials. Yeast-derived β-glucan particles (GPs) loaded with MC38 lysates and CpG form a sustained-release vaccine, triggering stronger antibody responses in murine models (75).

## 4 Adjuvants and administration routes of colorectal cancer vaccines

Adjuvants are substances that improve the efficiency of antigen presentation of APCs and enhance the immune response induced by vaccines. An appropriate administration route is another important part of amplifying the role of vaccines, improving accuracy and effectiveness, facilitating large-scale industrial production, and promoting the clinical transformation of vaccines.

### 4.1 Molecular adjuvants

Cytokines are major adjuvants that have been commercialized in colon cancer vaccines (39). GM-CSF, a white blood cell growth factor, is a secreting cytokine that provides robust immune potentiation through inducing activation of T cells and B cells while enhancing the production of IL-1, TNF, and IL-6 (76). It can be added directly to a vaccine or to a medium for maturation of DCs *in vitro*. Moreover, tumor cells are genetically modified to release GM-CSF. However, it is controversial that administration of excessive sustained doses of GM-CSF may induce myeloid suppressor cells, which deserves further clarification (77).

Another classical costimulatory strategy involves the application of TRICOM, a combination of three separate molecules found on APCs (78). B7.1 (CD80) is a protein that interacts with T-cell ligands CD28 and CTLA-4 resulting in T-cell stimulation *in vitro*. ICAM-A (CD56) is an adhesion molecule on the surface of APCs, binding to T-cell ligand LFA-A. LFA-3 (human CD58) is a surface protein that binds to CD2, priming T cells. TRICOM is a common companion of viral vector-based vaccines, widely investigated in many clinical trials (65, 79).

Other common adjuvants include IFN-γ and its upstream agonists, various toll-like receptor (TLR) agonists such as guanosine phosphate oligonucleotide (CPG), polyriboinosinic:polyribocytidylic acid (polyI:C), polyriboinosinic-polyribocytidylic acidpolylysine carboxymethylcellulose (polyI:CLC, best known as Hiltonol™) (80–82).

### 4.2 Administration routes of colorectal cancer vaccines

Subcutaneous, intramuscular, and intradermal vaccinations are among the most common administration routes of the CRC vaccine. The ingredients of vaccines reach lymph nodes through afferent lymph fluid and subsequently activate T-cell activation. Also, some DC-based vaccines are administered directly into the lymph node to present specific antigens to T cells.

*In situ* vaccination is an alternative route of administration in addition to standard subcutaneous and intravenous injections for the early diagnosis and treatment of CRC, which relies on endoscopy-guided puncture. *In situ* injection of K3-SPG as a monotherapy can fully induce systemic and persistent memory responses, and the combination of systemic administration of check point inhibitors (CPIs) and local administration of CD40 agonists has a synergistic antitumor effect (83).

Vaccination by the oral route is favored by populations as it is easy to administer, convenient, and needle-free. Liposomes or W/O/W double emulsions and biohybrid-bacterial hybridization have been made to encapsulate antigen peptides and TLR2 ligand Pam2Cys, which can activate mucosal immunity and reduce tumor burden in CRC murine models (84, 85). Outer membrane vesicles that exist in the complex gastrointestinal environment and cross the intestinal epithelial barrier are also worth further study as delivery systems of oral CRC vaccines (86).

## 5 Recent progression

### 5.1 Nanovaccines in colorectal cancer

**Figure 3:**
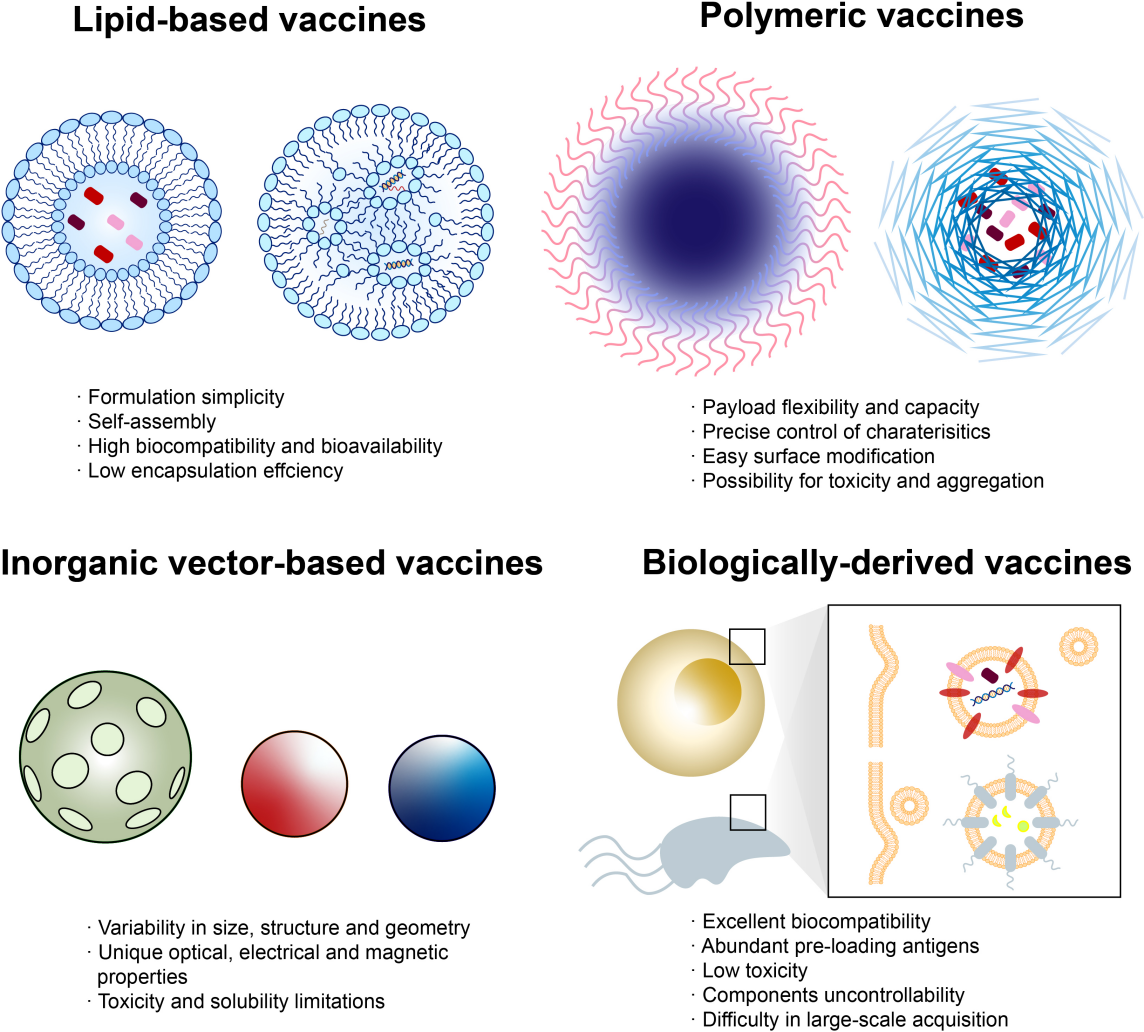
Classes of nanovaccines. Each class of nanovaccine features multiple subclasses, with some of the most common highlighted. Lipid-based vaccines include liposome-loading peptides (left) and lipid nanoparticle (NP)-loading nucleic acid (right). Polymeric vaccines include polymersome (left) that is able to load antigens inside the shell or directly onto the surface and polymer micelle (right)-wrapping peptides. Inorganic vectors include porous silica, gold NP, quantum dot, etc. Biologically derived vaccines include exosomes from human and outer membrane vesicles from microorganisms. Each class has numerous advantages and disadvantages regarding manufacturing, assembly, delivery, and patient response.

With the rapid development of material and biomedical science, new technologies have been provided to tailor cancer vaccines (87). Nanotechnology is one of the most promising candidates, possessing versatile properties such as multivalent delivery to lymphoid tissues and effective phagocytosis by APCs (88). Nanovaccines can be divided into four types: lipid-based vaccines, polymeric vaccines, inorganic vector-based vaccines, and biologically derived vaccines (89–91) (**Figure 3**).

Liposomes, composed of phospholipids, are capable of entrapping hydrophilic and lipophilic compounds. L-BLP25 (tecemotide), containing 25 amino acids from the MUC1 sequence as antigen and lipids as carrier, was investigated as an adjuvant therapy in a phase II trial involving patients with mCRC after R0/R1 resection of colorectal liver metastases (92). Median RFS and OS of the tecemotide arm were 6.1 months (95% CI 4.5–8.9, p = 0.1754) and 62.8 months (p = 0.2141), respectively, while improved median OS was observed in secondarily resected patients compared to two other trials, CELIM and FIRE-3 (93).

Polymers with various payloads and cargo-retention efficiency are ideal candidates as vaccine vectors, some precisely targeting the endosome for its characteristic pH/enzyme-responsiveness or acting as *in situ* cancer vaccine (94, 95). BanNV is a self-assembled vaccine based on maleimide-functionalized poly (ethylene oxide)-block-poly (d,l-lactic acid) (MAL-PEG-b-PLA) micelles, loading with neoantigen peptide (Adpgk) and dual synergistic adjuvants (96). PD-1 receptor can be sensitized by BanNV, resulting in 70% complete remission of neoantigen-specific cancer in combination with anti-PD-1 therapy in mice.

Although inorganic vectors such as gold, iron, and silica possess potential toxicity toward human, abundant physical properties are intriguing. For instance, the porous structure of silicon microparticles enables controlled release of tumor antigen and adjuvants inside, inhibiting CRC development in a murine model (97). A nanovaccine made up of both loaded with cancer cell membranes, CCM@(PSiNPs@Au), represents success in the combination of cancer vaccine and photothermal therapy (98). Chang et al. (99) successfully designed a Cu2O@CaCO3@HA nanovaccine that achieved synergistic CRC-targeted and TME-triggered photothermal/photodynamic/chemodynamic/calcium overload-mediated therapy in a CT26 murine model.

Biologically derived nanoparticles are vesicles extracted from outer membranes of microorganisms or human (exosome) with good biocompatibility and non-self-replicability (88). Contrary to prewrapped vaccines, a versatile antigen display platform for tumor vaccination was created by Cheng et al. (100). Targeted tumor antigens, such as Adgpk aimed at MC38 cells, can be displayed on the bioengineered outer membrane vesicle (OMV) through automatic formation of a peptide linkage after being tagged with specific proteins.

### 5.2 Neoantigen vaccines

Advances in sequencing technology and bioinformatics enlarge human multi-omic databases, allowing for the appropriate detection of neoantigens (2, 30). Comparing whole-genome (WGS) or whole-exome sequencing (WES) data of somatic tissues with those of germline tissues enables identification of shared mutations (29). Furthermore, RNA sequencing helps to infer the expression and activity of mutant peptides. HLA genotypes are also significant information for deterring the affinity of presentation and binding in subsequent steps (101). Computational approaches based on machine learning algorithms such as NetMHCpan, NetMHCIIpan, and MuPeXI predict and prioritize neopeptides (26).

The higher mutation frequency of CRC (102) especially for the MSI-H subgroup and the high-frequency and relatively fixed-mode mutations in microsatellite regions lead to the generation of shared multiple MHC I ligands (31). An off-the-shelf cancer vaccine, Nous-209, encodes several frameshift peptides and can be presented by human APCs, activating CD8+ T cells (103). Its combination with pembrolizumab is undergoing a phase I/II trial recruiting patients with metastatic gastrointestinal tumor (NCT04041310). Other clinical trials of neoantigen vaccines in CRC are currently underway (**Table 3**). Despite low TMB, MSS tumors can still produce a large number of tumor-specific HLA-I peptide ligands with high affinity through proteomics and polypeptide analysis based on CRC organoids (104).

**Table 3:** Major clinical trials of neoantigen vaccines in CRC.

NCT Number (status)	Tumor	Interventions	Adjuvant/combined therapy	Phases (Enrollment)	Completion Date
**NCT05141721** (Not yet recruiting)	mCRC	GRT-C901, GRT-R902	Fluoropyrimidine, oxaliplatin, bevacizumab, ipilimumab	Phase II/III (665)	March 2027
**NCT03639714** (Active, not recruiting)	MSS solid tumors include CRC	GRT-C901, GRT-R902	Nivolumab, Ipilimumab	Phase I/II (214)	March 2023
**NCT03953235** (Recruiting)	MSS solid tumors include CRC	GRT-C903,GRT-R904	Nivolumab, Ipilimumab	Phase I/II (144)	December 2023
**NCT04912765** (Recruiting)	mCRC, hepatocellular cancer	Neoantigen Dendritic Cell Vaccine	Nivolumab	Phase II (60)	May 2025
**NCT05243862** (Not yet recruiting)	mCRC	PolyPEPI1018	Montanide ISA 51, Atezolizumab	Phase II (28)	March 2026
**NCT05078866** (Not yet recruiting)	Lynch Syndrome	GAd-209-FSP, MVA-209-FSP	/	Phase Ib/II (45)	December 2025
**NCT01885702** (Active, not recruiting)	Colorectal Cancer (MSI) or Lynch syndrome	DC vaccination	/	Phase I/II (25)	December 2022
**NCT05130060** (Recruiting)	mCRC	PolyPEPI1018	Montanide ISA 51, TAS-102	Phase I (15)	May 2024
**NCT04117087** (Recruiting)	MSS mCRC, Pancreatic Cancer	KRAS peptide vaccine	Poly-ICLC / nivolumab, ipilimumab.	Phase I (30)	June 2024
**NCT04853017** (Recruiting)	KRAS/NRAS mutated (G12D or G12R) solid tumor (including CRC)	ELI-002 2P	Amph-CpG-7909	Phase I (18)	September 2024
**NCT04147078** (Recruiting)	Solid tumors including CRC	Neoantigen-primed DC Vaccine	/	Phase I (80)	June 2023
**NCT04799431** (Not yet recruiting)	mCRC, metastatic pancreatic cancer	Neoantigen Vaccine	Poly-ICLC/ Retifanlimab	Phase I (12)	February 2026

mCRC, metastatic colorectal cancer; MSS, microsatellite stable; pMMR, proficient mismatch repair.

## 6 Conclusion and future perspective

Cancer vaccines for CRC have gone a long way, and essential progress has been made. So far, present research has indicated that therapeutic vaccines appear to be suitable for cancer patients with minimal lesion residue or those at advanced stages as an adjuvant therapy. In order to break Programmed Cell Death-Ligand 1 (PD-L1)/PD-1 axis, a significant negative feedback loop restricting tumor immunity, the combination of CRC vaccines and anti-PD-1 drugs is frequently tested in clinical trials, demonstrating promising effects on patients who would not benefit from either therapy alone.

However, limited evidence of clinical benefits has been observed despite the successful induction of immune response. Despite the initial success of sipuleucel-T, further vaccines have failed to progress and there has been limited uptake of sipuleucel-T in the clinic probably due to its limited effect on prolonging OS of patients and high costs of production (105). In addition, various types of CRC vaccines have been evaluated in clinical trials, but none has led to significant benefits in large phase III trials. A possible explanation of the phenomenon is that the effective antitumor immunity does not last long enough to improve the survival of patients. Additionally, amplification of both immunogenic and tolerogenic T-cell subclones may nullify the therapeutic effects (106). There are still several obstacles with CRC vaccines. Firstly, immunosuppression/immune tolerance is a critical problem resisting vaccines to prolong the survival of CRC patients. According to the “immunoediting” theory, the paradoxical interaction between tumor cells and the immune system depicts a sequential course of elimination, equilibrium, and escape (107). An immunosuppressive microenvironment at the “escape” phase cannot be easily converted to an antitumor one, crippling anticipated effects of efficiently eradicating tumor cells. Secondly, there is always a trade-off between precise medication and off-the-shelf large-scale production. In terms of neoantigen vaccines, both the cost and time of prevaccination procedures require reduction, especially for patients with metastatic disease. Currently, the period for tissue acquisition to personalized vaccine delivery varies from 3 to 5 months (108). Moreover, the early-stage diagnosis rate of CRC or precancer lesions raises the question of whether prophylactic vaccines for non-viral origin cancers are a dream or a real possibility. Cancer vaccines targeted at MUC1 that were proven safe and elicited tumor-specific long-term memory in clinical trials are under consideration for preventative purposes (109, 110). However, how strong this immunity will be and how long it will persist are crucial points that deserve further investigation.

Future clinical trials will be urged to carry out in stratified patient populations. Several trials are exploring the safety and effect of prophylactic vaccines in patients diagnosed with Lynch syndrome (NCT05078866). Rapid development of neoantigens and nanovaccines also sheds new light into the field, making CRC vaccines a proud member of the immunotherapy family.

## Author contributions

WJ, TZ contribute equally to this work. All authors contributed to the article and approved the submitted version.

## Funding

This study was supported by the Shanghai Science and Technology Commission, 18ZR1424300 (RZ); Shanghai Hospital Development Center, SHDC2020CR1026B (RZ); Shanghai Health Commission, 2019SY058 (RZ); National Natural Science Foundation of China, 82002475 (XC); Shanghai Sailing Program, 20YF1427700 (XC).

## Conflict of interest

The authors declare that the research was conducted in the absence of any commercial or financial relationships that could be construed as a potential conflict of interest.

## Publisher’s note

All claims expressed in this article are solely those of the authors and do not necessarily represent those of their affiliated organizations, or those of the publisher, the editors and the reviewers. Any product that may be evaluated in this article, or claim that may be made by its manufacturer, is not guaranteed or endorsed by the publisher.
